# Creation of a nanoformulated cabotegravir prodrug with improved antiretroviral profiles

**DOI:** 10.1016/j.biomaterials.2017.10.023

**Published:** 2017-10-15

**Authors:** Tian Zhou, Hang Su, Prasanta Dash, Zhiyi Lin, Bhagya Laxmi Dyavar Shetty, Ted Kocher, Adam Szlachetka, Benjamin Lamberty, Howard S. Fox, Larisa Poluektova, Santhi Gorantla, JoEllyn McMillan, Nagsen Gautam, R. Lee Mosley, Yazen Alnouti, Benson Edagwa, Howard E. Gendelman

**Affiliations:** a Department of Pharmaceutical Sciences, University of Nebraska Medical Center, Omaha, NE 68198, USA; b Department of Pharmacology and Experimental Neuroscience, University of Nebraska Medical Center, Omaha, NE 68198, USA; c Nebraska Nanomedicine Production Plant, University of Nebraska Medical Center, Omaha, NE 68198, USA

**Keywords:** Cabotegravir, Long-acting, Prodrug, Nanoformulation, HIV-1

## Abstract

Long-acting parenteral (LAP) antiretroviral drugs have generated considerable interest for treatment and prevention of HIV-1 infection. One new LAP is cabotegravir (CAB), a highly potent integrase inhibitor, with a half-life of up to 54 days, allowing for every other month parenteral administrations. Despite this excellent profile, high volume dosing, injection site reactions and low body fluid drug concentrations affect broad use for virus infected and susceptible people. To improve the drug delivery profile, we created a myristoylated CAB prodrug (MCAB). MCAB formed crystals that were formulated into nano-particles (NMCAB) of stable size and shape facilitating avid monocyte-macrophage entry, retention and reticuloendothelial system depot formulation. Drug release kinetics paralleled sustained protection against HIV-1 challenge. After a single 45 mg/kg intramuscular injection to BALB/cJ mice, the NMCAB pharmacokinetic profiles was 4-times greater than that recorded for CAB LAP. These observations paralleled replicate measurements in rhesus macaques. The results coupled with improved viral restriction in human adult lymphocyte reconstituted NOD/SCID/IL2Rγc^−/−^ mice led us to conclude that NMCAB can improve biodistribution and viral clearance profiles upon current CAB LAP formulations.

## 1. Introduction

Following the 1983 discovery of the human immunodeficiency virus type one (HIV-1), remarkable progress was made in the development of effective diagnostics and treatments [1]. Arguably, the most successful of all is effective antiretroviral therapy (ART). ART has markedly reduced disease-associated morbidities and mortality, enabling a nearly normal quality of life for infected people [2,3]. Nevertheless, life-long treatment is still required to suppress viral replication and contain disease. HIV-1 resistance [4,5], drug toxicities [6,7], and poor patient adherence [8] have also impeded therapeutic effectiveness. Treatment fatigue, lack of financial and social support, co-existing mental symptoms, and substance abuse can result in lack of adherence to ART regimens [9]. Therefore, ways to improve regimen adherence are greatly needed.

An important milestone in recent years to improve ART adherence is long-acting parenteral (LAP) antiretroviral drugs (ARVs) [10]. Changes in treatment patterns from daily oral to monthly or even less-frequent administration may also provide greater patient privacy and satisfaction [11,12]. A survey conducted in 400 HIV-1 seropositive patients indicated that 73% of patients would consider switching from daily pill regimens to LAP regimens [13]. However, not all ARVs can be redeveloped as LAPs based on drug potency and physicochemical properties. In fact, only a few of ARV candidates have been advanced to clinical studies. Cabotegravir (CAB) plus rilpivirine (RPV) is first-ever long-acting combination ART regimen. Monthly or every other month CAB and RPV LAP formulations demonstrated comparable antiretroviral activity to daily oral three-drug combinations for maintenance therapy [14]. CAB is a novel integrase strand transfer inhibitor (INSTI) with low aqueous solubility, high melting point, high potency, long half-life, and slow metabolic clearance [15,16]. These properties enable CAB to be formulated in a 200-mg/mL suspension (CAB LAP) and administered intramuscularly monthly or even less frequently [14,17]. An additional benefit rests in that CAB is primarily metabolized by uridine diphosphate glucuronosyltransferase (UGT) 1A1, with low potential to interact with other ARVs [16,18]. CAB LAP has also proven to be highly protective against rectal, vaginal, and intravenous SHIV transmission in non-human primates [19–22], and has been advanced into clinical trials for HIV prevention (NCT02720094). Despite such promising drug profiles, dosing pattern has limitations. Specifically, split injections given in 2 mL volumes are required to achieve 800-mg doses, leading to treatment cessations because of intolerable injection site reactions [14,23]. However, even with the high injection volumes, the maximal dosing interval is only 8 weeks. Recently in a phase 2a study investigating safety and tolerability of CAB LAP in HIV-uninfected men (ECLAIR; NCT02076178) [23], 800 mg dose every 12 weeks was selected based on previous clinical studies [17,24], aiming to maintain plasma CAB concentrations above 4 times protein-binding-adjusted 90% inhibitory concentration (4 × PA-IC_90_, 660 ng/mL), a concentration demonstrated to be protective against new infections in macaques [19–22]. However, two-thirds of participants had faster than anticipated drug absorption from the injection site leading to plasma drug concentrations below targeted concentration of 4 × PA-IC_90_ at 12 weeks. Therefore, follow up study HPTN 083, a study for pre-exposure prophylaxis (PrEP) in HIV-uninfected cis gender men and transgender women, will require shortened dosing intervals (600 mg in 3 mL injection volumes every 8 weeks) to achieve effective protection with relevant antiretroviral plasma drug concentrations. Taken together, a means to extend the dose interval beyond 12 weeks and reduce injection volumes could bring broader usage to such regimens [25].

Progress in medicinal chemistry and nanotechnology has provided new opportunities to improve ARV delivery. Diverse strategies, such as chemical modification, cell-based drug delivery, HIV reservoir targeting, alternative delivery methods, are being tested to improve ARV pharmacokinetic (PK) profiles and biodistribution (BD) [26–28]. Modifications in ARV structures and delivery platforms can facilitate drug depot formation and lymphoid targeting [29–31], and have the potential to better penetrate HIV reservoirs, lower drug dosing, and reduce systemic toxicities [31–33]. To these ends, we designed a CAB nanoformulated prodrug, aiming to improve the drug’s lipophilicity, hydrophobicity, and cellular entry and retention to improve the drug’s ability to access anatomical reservoirs while maintaining high plasma concentrations. The result was a nanoformulated myristoylated CAB (NMCAB) with tailored formulation modifications that enhance tissue access and improve its antiretroviral activities.

## 2. Materials and methods

### 2.1. Materials

Native CAB and CAB LAP (200 mg/mL) were graciously obtained from ViiV Healthcare (Research Triangle Park, NC, USA). Myristoyl chloride, poloxamer 407 (P407), *N*,*N*-diisopropylethylamine (DIEA), dimethylformamide (DMF), 1-octanal, ciprofloxacin, paraformaldehyde (PFA), and 3,3′-diaminobenzidine (DAB) were purchased from Sigma-Aldrich (St. Louis, MO, USA). Dulbecco’s Modified Eagle’s Medium (DMEM) was purchased from Corning Life Sciences (Tewksbury, MA, USA). Heat-inactivated pooled human serum was purchased from Innovative Biologics (Herndon, VA, USA). Cell counting kit-8 (CCK-8) was purchased from Dojindo Molecular Technologies, Inc. (Rockville, MD, USA). Gentamicin, HPLC grade acetonitrile (ACN), HPLC grade methanol, and Optima grade LC/MS water was purchased from ThermoFisher Scientific (Waltham, MA, USA). FITC mouse anti-human CD45, Alexa Fluor 700 mouse anti-human CD3, APC mouse anti-human CD4, and BV421 mouse anti-human CD8 were purchased from BD Biosciences (San Jose, CA, USA). Monoclonal mouse anti-human HIV-1p24 (clone Kal-1), monoclonal mouse anti-human leukocyte antigen (HLA-DR; clone CR3/43), and the polymer-based HRP-conjugated anti-mouse EnVision + secondary were purchased from Dako (Carpinteria, CA, USA).

### 2.2. Myristoyl CAB (MCAB) synthesis

**Fig. 1:**
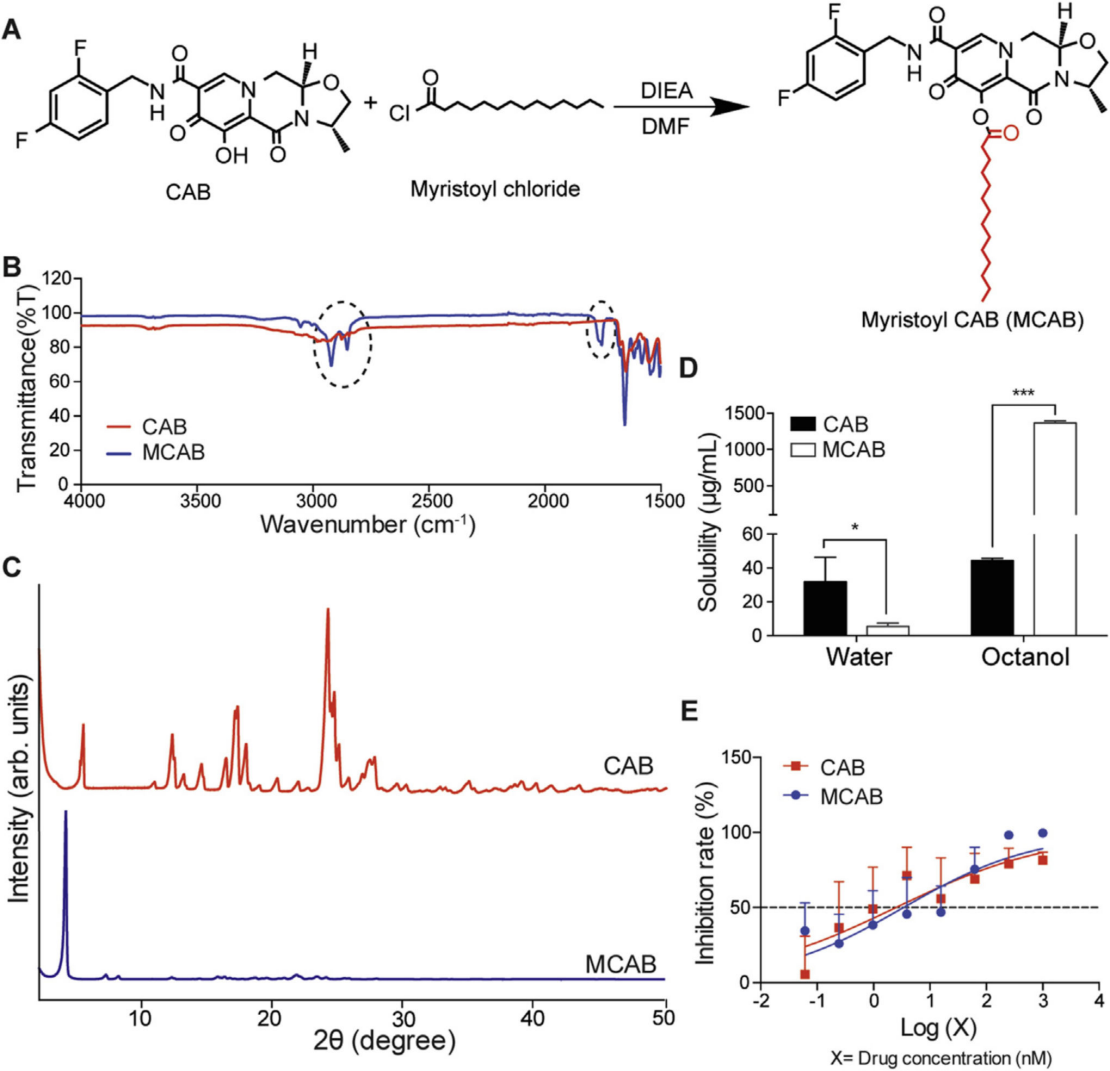
MCAB synthesis and characterization (**A**) Myristoylated CAB (MCAB) was synthesized with a final yield of 85%. (**B**) Fourier transform infrared (FT-IR) spectroscopy was performed to compare CAB and MCAB. Circled absorption bands at 1760, 2855 and 2923 cmr^−1^ are associated with the conjugated fatty acid chain for MCAB. (**C**) X-ray diffraction (XRD) analysis of MCAB and CAB illustrated more uniform crystalline for MCAB as compared to native CAB. (**D**) Solubilities of CAB and MCAB in water and 1-octanol were measured at room temperature. Compared to CAB, MCAB showed a five-fold decrease in aqueous solubility, and a 30-fold increase in 1-octanol solubility. Data are expressed as mean ± SD for *n* = 4 samples per group evaluated. The means were compared by two-tailed Student’s *t*-test. *P< 0.05, ***P< 0.001. (**E**) Antiretroviral activities of CAB and MCAB were evaluated in HIV-1_ADA_ infected human monocyte derived macrophages (MDM). Inhibition rate was presented as percentage of reduced HIV reverse transcriptase (RT) activity as compared to positive control RT activity. Nonlinear regression was performed using log (drug concentration) vs. Inhibition rate with variable slope, and drug concentration for 50% inhibition (IC_50_) was calculated. IC_50_ values were 2.5 nM and 3.4 nM for CAB and MCAB, respectively. Data are expressed as mean ± SD for *n*= 6 samples per group.

Synthesis of MCAB was conducted according to the scheme shown in Fig. 1A. Briefly, a precooled (0 °C) solution of CAB (4.9 mM, 1 equivalent) in anhydrous DMF (35 mL) was deprotonated using DIEA (9.8 mM, 2 equivalents) and then reacted with myristoyl chloride (9.8 mM, 2 equivalents) for 16 h at room temperature. After completion of the reaction, the mixture was concentrated and the product isolated by flash silica gel chromatography using a mixture of ethyl acetate and hexane (4:1). The fractions containing UV active MCAB were dried and washed with diethyl ether, followed by drying under vacuum to get the final product.

### 2.3. MCAB physicochemical characterizations

Proton nuclear magnetic resonance (^1^H NMR) spectra of CAB and MCAB were recorded on a Varian Unity/Inova-500 NB (500 MHz; Varian Medical Systems Inc., Palo Alto, CA, USA). ^1^H NMR data is reported in parts per million (ppm) downfield from tetra-methylsilane as an internal standard. Fourier transform infrared spectroscopy (FT-IR) analysis was performed using a Spectrum Two FT-IR spectrometer (PerkinElmer, Waltham, MA, USA). Comparative crystallographic analyses of CAB and MCAB by powder X-ray diffraction (XRD) were carried out in the 2θ range of 2–50° using PANalytical Empyrean diffractometer (PANalytical Inc., West-borough, MA, USA) with Cu-Kα radiation (1.5418 Å) at 40 kV, 45 mA setting. A mask of 20 mm and a divergence slit of 1/8° were used on the incident beam path. A nickel foil filter was used to eliminate the diffraction peaks due to possible K_b_ wavelength.

### 2.4. Solubility test

The solubilities of CAB and MCAB in water and 1-octanol were determined by adding excess drug to each solution at room temperature then mixing for 24 h. Samples were centrifuged at 20,000 × g for 10 min to pellet insoluble drug. The supernatants containing solubilized drug were vacuum-dried then re-dispersed in methanol for drug concentration measurement using a Waters ACQUITY ultra performance liquid chromatography (UPLC) H-Class System with TUV detector and Empower 3 software (Milford, MA, USA). CAB and MCAB samples were separated on a Phenomenex Kinetex 5 µm C18 column (150 × 4.6 mm) (Torrance, CA) using either 65% 5.0 mM KH_2_PO_4_, pH 3.2/35% ACN or 90% ACN/10% water with a flow rate of 1.0 mL/min and detected at 254 and 230 nm, respectively. Drug content was quantitated by comparison of peak area to those of known standards (0.05–50 µg/mL in methanol).

### 2.5. Nanoformulated MCAB (NMCAB) manufacture and characterization

NMCAB and nanoformulated CAB (NCAB) were manufactured by high-pressure homogenization (Avestin EmulsiFlex-C3; Avestin Inc., Ottawa, ON, Canada). Briefly, MCAB or CAB (5% w/v) was pre-mixed in a P407 solution (0.5% w/v in endotoxin free water) for 16 h at room temperature followed by homogenization at 20,000 psi until the desired particle size of approximately 300 nm was achieved [34]. Effective diameter (D_eff_), polydispersity index (PdI), and ζ-potential were assessed by dynamic light scattering (DLS) using a Malvern Zetasizer Nano Series Nano-ZS (Malvern Instruments, Westborough, MA, USA). Particle morphology was determined using a Hitachi S4700 field emission scanning electron microscope (SEM) (Hitachi High Technologies America Inc, Schaumburg, IL, USA). The stability of NMCAB was monitored at 4°C for up to 3 months in terms of D_eff_, PdI and ζ-potential. The crystalline structures of lyophilized CAB LAP and NMCAB were determined by XRD and compared against CAB or MCAB as described above. Drug loadings and encapsulation efficiencies were calculated using the following equations:

(1) $$\text{Drug Loading}(\%)=\frac{\text{weight of drug in formulation}}{\text{weight of lyophilized formulation}}\times 100$$

(2) $$\text{Encapsulation efficiency}(\%)=\frac{\text{weight of drug in formulation}}{\text{weight of drug fed initally}}\times 100$$

### 2.6. Cell models to assess antiretroviral activity

Monocyte-derived macrophages (MDM) were utilized. Human peripheral blood monocytes were obtained and cultured as previously described [35,36]. Briefly, monocytes were obtained by leukapheresis of HIV-1/2 and hepatitis B seronegative donor blood cells, followed by purification via countercurrent centrifugal elutriation. Elutriated monocytes were cultured as adherent cells in DMEM supplemented with 10% heat-inactivated pooled human serum, 10 µg/mL ciprofloxacin, 50 µg/mL gentamicin, and 1000 U/mL recombinant macrophage colony stimulating factor. Cells were maintained at 37 °C in a 5% CO_2_ incubator. Seven days later, differentiated macrophages were treated with various concentrations (0.06–1000 nM) of native CAB or MCAB for 30 min, followed by HIV-1_ADA_ challenge at a multiplicity of infection (MOI) of 0.1 infectious viral particles/cell. Four hrs after challenge, cells were washed three times with sterile phosphate-buffered saline (PBS) followed by incubation with the same concentration of each compound used before infection. Cells were cultured for additional 7 days with half-media changes every other day with DMEM media containing the same drug concentrations. Supernatants were collected 7 days after the challenge for HIV reverse transcriptase (RT) activity determination as previously described [36,37]. To determine the antiretroviral activity of nanoformulations, MDM were treated with NMCAB, CAB LAP, or NCAB containing 100 µM drug for 8 h, followed by 3 PBS washes to remove any extracellular drug. At predetermined time points (days 0, 2, 5, 10, and 15), MDM were challenged with HIV-1_ADA_ at an MOI of 0.1 for 4 h. Seven days after the virus challenge, culture media were analyzed for RT activity, while adherent MDM were fixed with 4% PFA and HIV-1p24 protein expression was assessed by immunocytochemistry [30].

### 2.7. Transmission electron microscopy (TEM)

The morphology of macrophages loaded with nanoformulations was imaged by TEM. For uptake, MDM were treated with NMCAB or CAB LAP for 8 h. For retention, after 8 h drug loading, MDM were washed 3 times with PBS then cultured in fresh media for an additional 48 h. For cell sample collection, adherent MDM were washed with PBS for 3 times and then scraped into PBS and pelleted at 1000 × g for 8 min. Muscle tissue from BALB/cJ mice at sites of injection were also imaged by TEM. Twenty-four hrs after an intramuscular injection of NMCAB or CAB LAP at 45 mg CAB equivalents/kg, mice were sacrificed and muscle tissues at the site of injection were dissected into 1–2 mm^3^ cubes for TEM sample processing. Both cell and tissue samples were fixed by immersion in a solution of 2% glutaraldehyde, 2% paraformaldehyde in 0.1M Sorenson’s phosphate buffer (pH 6.2), followed by post-fixation in 1% osmium tetroxide for 60 min then dehydration in a graded ethanol series (50, 70, 90, 95, 100%). Subsequently, samples were incubated in 50:50 mixture of a propylene oxide:araldite resin solution for 16 h. This was performed to evaporate propylene oxide and was followed by an additional 2 h incubation with fresh resin at room temperature prior to embedding (65 °C for 24 h). Sections of 100 nm were prepared with Leica UC6 Ultracut ultramicrotome, placed on 200 mesh copper grids, and stained with 2% uranyl acetate, followed by Reynolds Lead Citrate. Grids were examined on a Tecnai G2 Spirit TWIN (FEI) transmission electron microscope (Hillsboro, OR, USA) operated at 80 kV and images were acquired digitally with an AMT digital imaging system (Woburn, MA, USA).

### 2.8. Pharmacokinetics (PK) and biodistribution (BD) of NMCAB in BALB/cJ mice

Male BALB/cJ mice (Jackson Labs, Bar Harbor, ME, USA) were dosed intramuscularly with NMCAB or CAB LAP 45 mg CAB equivalents, followed by weekly blood collection in heparinized tubes via cheek bleeding. Plasma was collected via centrifugation at 2000 × g for 5 min for the drug quantitation by ultra-performance liquid chromatography tandem mass spectrometry (UPLC-MS/MS) (see supplementary method). At day 28 and day 58 after nano-formulation injection, 5 mice in each treatment group were sacrificed and tissues including liver, lung, spleen, lymph node were collected for drug quantitation by UPLC-MS/MS (see supplementary method). To identify potential drug depots for NMCAB, PK at early time points was assessed in male BALB/cJ mice after a single intramuscular injection of NMCAB or CAB LAP (45 mg CAB equivalents/kg). At predetermined time points (15 min, 1, 2, 4, and 8 h and 1, 3, 7, 10 and 14 days), 25 mL of whole blood was collected and levels of both CAB and MCAB were determined. Tissues were collected at days 1, 3, 7 and 14 for drug quantitation by UPLC-MS/MS.

### 2.9. NMCAB PK in rhesus macaques

Two male Chinese rhesus macaques (PrimeGen; 3 years old; 4.0 and 4.7 kg) were anesthetized with 10 mg/kg ketamine and injected intramuscularly with NMCAB at 45 mg CAB equivalents/kg in 2.1 and 2.5 mL, respectively. NMCAB was manufactured by the Nebraska Nanomedicine Production Plant Good Laboratory Practices (GLP) laboratory (See supplementary method). Blood was collected into potassium EDTA tubes before drug administration, and at days 4, 7, 11, and 18 after the administration, and biweekly thereafter. Plasma was obtained for CAB and MCAB drug quantitation and metabolic panels, while peripheral blood mononuclear cells (PBMCs) were obtained for complete blood counts. These were performed by the Nebraska Medical Center Pathology and Microbiology laboratory.

### 2.10. PK parameters analyses

Non-compartmental PK analyses for plasma CAB were performed using WinNonlin-5.1 (Certara USA, Inc., Princeton, NJ, USA) for both BALB/cJ mice and rhesus macaques studies.

### 2.11 Studies of viral restriction in humanized adult lymphocyte mice

Male 6–8-week-old NOD/SCID/IL2Rγc^−/−^ (NSG) mice (Jackson Labs, Bar Harbor, ME, USA) were injected intramuscularly with NMCAB or CAB LAP at 45 mg CAB equivalents/kg. Eleven days after nanoformulation treatments, mice were reconstituted by intraperitoneal injection with 25 × 10^6^ human peripheral blood lymphocytes (PBL) obtained by leukapheresis and centrifugal elutriation. Eleven days after reconstitution, mice were challenged with 10^4^ 50% tissue culture infectious dose (TCID_50_) HIV-1_ADA_ by intraperitoneal injections. Mice were sacrificed 10 days after viral challenge. The experimental timeline is shown in Fig. 7A. Peripheral blood was collected at days 10 (prior to PBL reconstitution), 21 (prior to HIV-1 challenge), and 32 (10 days post HIV-1 challenge) for flow cytometry analysis of human pan-CD45, CD3, CD4 and CD8 immune markers [38]. Plasma was collected via centrifugation at 2000 × g for 5 min for drug quantitation by UPLC-MS/MS. HIV-1 RNA was analyzed in day 32 plasma samples using the Roche Amplicor and Taqman-48 system with HIV-1 kit V 2.0 according to the manufacturer’s instructions (Roche Diagnostics, Indianapolis, USA). Tissues were collected for CAB concentrations by UPLC/MS/MS, viral RNA and DNA quantitation by semi-nested real-time PCR [39], and immunohistochemical staining for HIV-1p24 antigen as described previously [40].

### 2.12. Statistics

For all *in vitro* studies, data were presented as mean ± standard deviation (SD), and experiments were preformed using a minimum of three biological distinct replicates. For *in vivo* studies, data were presented as mean ± standard error of the mean (SEM), each group contained 5 (PK studies) or 8 (viral restriction study) mice. For comparison of two groups, Student’s *t*-test (two-tailed) was used. For comparison among three groups, one-way ANOVA followed by Tukey’s or Dunnett T3 post hoc test was used based on the homogeneity of the samples. No outliers from animal or cell experiments were excluded. *P* < 0.05 was considered to be significant (*P < 0.05; **P < 0.01; ***P < 0.001). All the data were analyzed using GraphPad Prism 6.0 software (La Jolla, CA) or IBM SPSS (Armonk, NY, USA).

### 2.13. Study approval

All animal studies were approved by the University of Nebraska Medical Center Institutional Animal Care and Use Committee in accordance with the standards incorporated in the Guide for the Care and Use of Laboratory Animals (National Research Council of the National Academies, 2011). Human monocytes and PBL were isolated by leukopheresis from HIV-1/2 and hepatitis seronegative donors according to an approved UNMC IRB exempt protocol.

## 3. Results

### 3.1 Synthesis and characterization of myristoylated CAB (MCAB)

A fourteen-carbon fatty acid chain modified CAB (MCAB) was synthesized (Fig. 1A) with a final yield of 85%. The chemical structure of MCAB was characterized by ^1^H NMR and FT-IR spectra and compared to CAB as shown in Fig. S1 and Fig. 1B. Triplets at 0.88, 1.77 and 2.69 ppm and a broad singlet at 1.26 ppm correspond to the terminal methyl and repeating methylene protons of the fatty acid alkyl chain in MCAB. Disappearance of the phenol proton peak at 11.5 ppm confirmed the substitution of CAB’s hydroxyl proton with myristoyl moiety. In addition, absorption bands at 2855 and 2923 cm^−1^ in the FT-IR spectrum associate with the C-H stretches of the fatty acid chain (Fig. 1B). A band at 1760 cm^−1^ is attributed to the carbonyl C═O stretch of the ester bond present in the fatty acid chain. Comparative crystallographic analyses of CAB and MCAB by XRD revealed more uniform crystals in MCAB (Fig. 1C). Myristoylation reduced water solubility from 31.9 ± 14.4 µg/mL (CAB) to 5.6 ± 1.8 µg/mL (MCAB), and increased solubility in 1-octanol by 30-fold from 44.4 ± 1.3 µg/mL (CAB) to 1366.5 ±27.5 µg/mL (MCAB) (Fig. 1D). HIV RT activity measurements demonstrated that the IC_50_ of MCAB (3.1 nM) and CAB (2.5 nM) against HIV-1_ADA_ in MDM were comparable (Fig. 1E), indicating that the chemical modification of CAB did not alter its antiretroviral activities.

### 3.2. Nanoformulated MCAB (NMCAB)

**Fig. 2:**
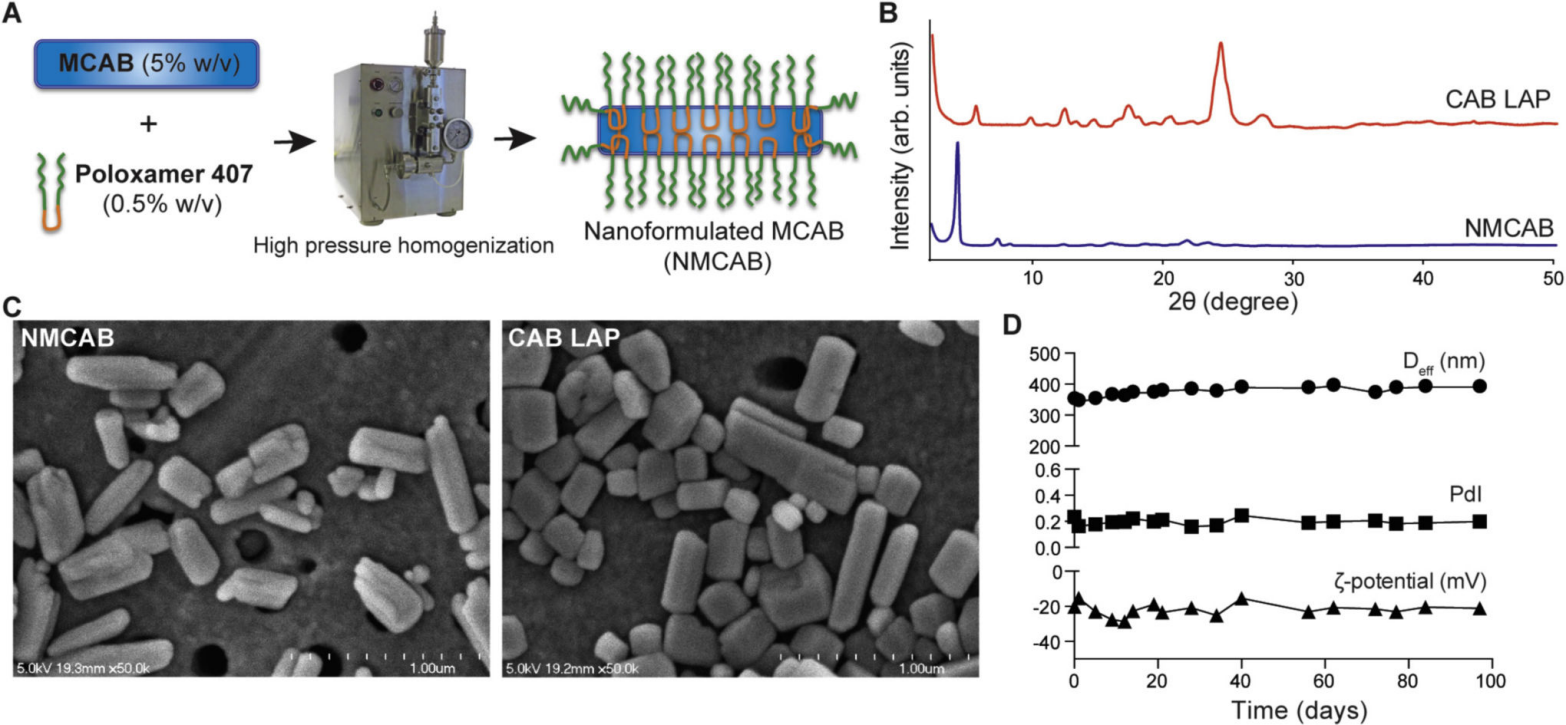
Nanoformulated MCAB (NMCAB) preparation and physicochemical characterization (**A**) NMCAB nanoparticles were prepared by high-pressure homogenization. (**B**) XRD analysis was performed on lyophilized CAB LAP and NMCAB formulations. (**C**) Morphologies of NMCAB and CAB LAP were visualized by scanning electron microscopy (SEM). Scale bar: 1 µm. (**D**) Time course measurements of NMCAB stability over 90 days for intensity-mean Z-averaged particle diameter (D_eff_), ζ-potential, and polydispersity index (PdI) of NMCAB stored at 4 °C. Data are expressed as mean ± SD for *n*= 3 measurements.

NMCAB was manufactured successfully by high-pressure homogenization using a drug to polymer ratio of 10:1 (w/w) as outlined in Fig. 2A. There were two principal controls to evaluate the effectiveness of NMCAB. The first was CAB LAP, which is the formulation now being used in Phase III clinical trials. The second was native CAB formulated in the same excipient (P407) used for NMCAB. This formulation is referred to as nanoformulated CAB (NCAB). NCAB was manufactured under the same conditions as NMCAB but without the chemical modifications made to the drug itself. Both CAB LAP and NCAB were used as assay controls. XRD crystallographic analyses (Fig. 2B) illustrated unchanged diffractograms for both NMCAB and CAB LAP formulations as compared to the unformulated MCAB and CAB (Fig. 1C). This indicated that drug encapsulation with high-pressure homogenization did not interfere with its crystal integrity. Intensity-mean Z-averaged particle diameter (D_eff_), ζ-potential, and polydispersity index (PdI) of formulations were determined by DLS (Table S1). NMCAB and NCAB were comparable in particle size (318 ± 25 and 315 ± 26 nm respectively) with PdI < 0.3 and negative surface charges. CAB LAP exhibited a size of 257 ± 6 nm, and similar PdI and ζ-potential with NMCAB. Due to the limited amount of excipient usage, high drug loading (90 and 86%) and encapsulation efficiencies (91 and 63%) were achieved for NMCAB and NCAB. Rod shape structure, a preferred morphology for macrophage uptake [41–43], was observed by scanning electron microscopy (SEM) for both NMCAB and CAB LAP (Fig. 2C). NMCAB was stable with consistent PdI and ζ-potential with minimal increases in D_eff_ (39 nm) after storage at 4 °C for up to three months (Fig. 2D).

### 3.3. MDM viability, uptake and retention

**Fig. 3:**
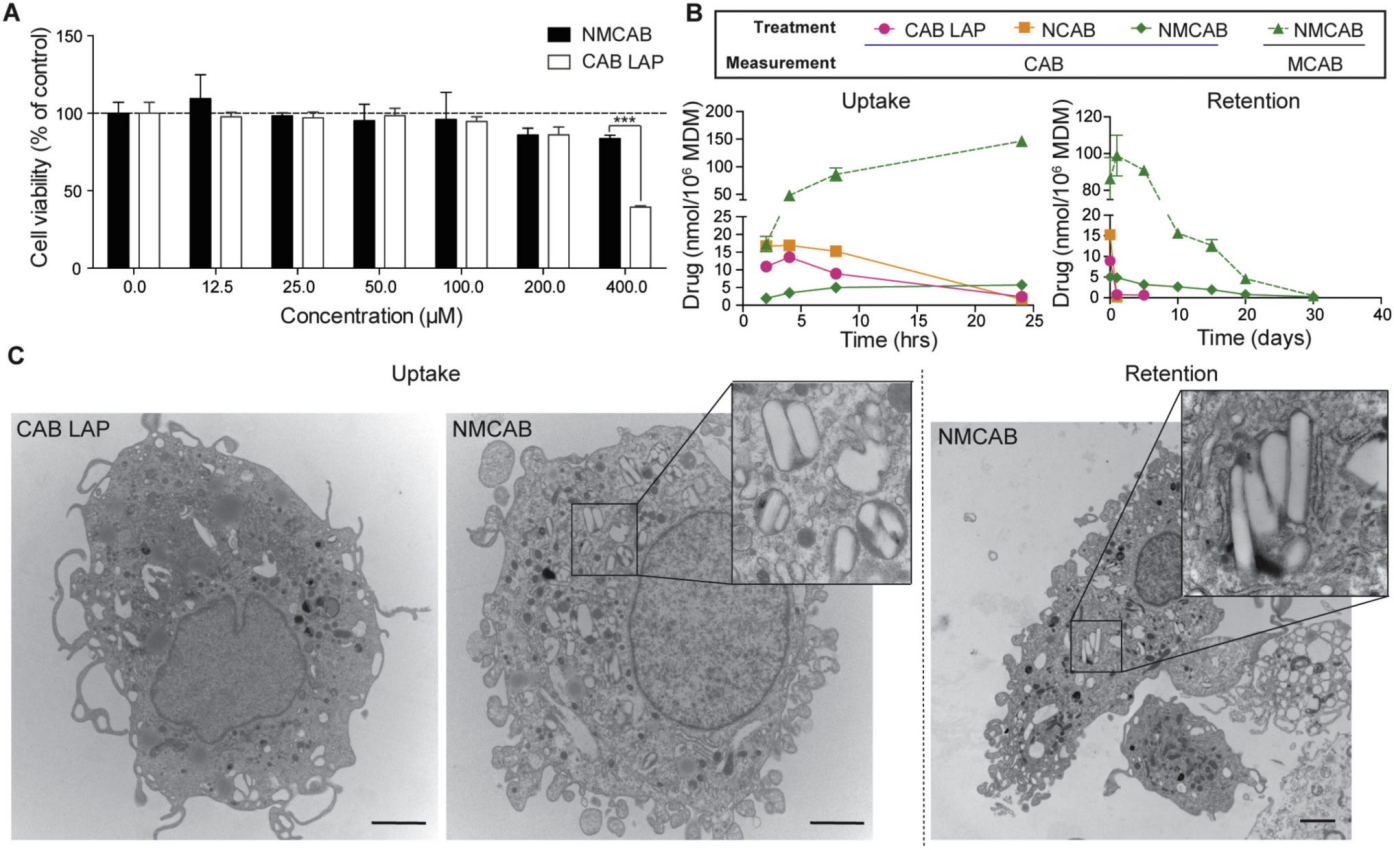
NMCAB enhances drug uptake and retention in MDM (**A**) Cell viability of MDM was assessed 8h after NMCAB or CAB LAP treatment over a concentration range of 0–400 µM. Results are shown as percentage of cell viability as compared to untreated MDM. Data are represent mean ± SD for *n* = 3 samples per group. Comparisons between NMCAB and CAB LAP were performed by Student’s *t*-test. ***P< 0.001. (**B**) NMCAB uptake and retention were assessed in human MDM compared to nanoformulated CAB (NCAB) prepared using identical excipient and condition as NMCAB, and CAB LAP. For uptake, intracellular drug concentrations were determined in MDM receiving 100µM NMCAB, NCAB or CAB LAP treatment for 2–24h. Drug retention in MDM was determined after an 8-h drug loading followed by washing and culturing with fresh media for additional 1–30 days. Intracellular drug concentrations were analyzed by HPLC-UV/Vis. Both MCAB (dashed lines) and CAB (solid lines) were measured independently for NMCAB treated cells. Data are expressed as mean ± SD for *n* = 3 samples per group. (**C**) Transmission electron microscopy (TEM) was performed to visualize morphologies of formulation-loaded MDM. MDM were imaged after 8h incubation with CAB LAP or NMCAB containing 100µM of drug (Uptake). Drug crystals were observed only in NMCAB treated MDM. Retained crystals in NMCAB treated MDM were observed after a wash-off period of 48 h (Retention). Scale bar: 2 µm.

Cell viability assays revealed that both NMCAB and CAB LAP treatment had no effect on MDM viability with drug concentrations of up to 100 µM. Treatment with formulations containing 200 µM drug slightly reduced cell viability to 85.9% (NMCAB) and 86.1% (CAB LAP), respectively. There was no statistical difference between the two treatment groups. However, 400 µM CAB LAP treatment reduced MDM cell viability to 39.4% compared to 83.7% in NMCAB treated cells (*P* < 0.001) (Fig. 3A). Based on this result, 100 µM was selected for all cell culture studies. NMCAB was readily taken up by MDM and intracellular concentration increased over time to 146 nmoles/10^6^ cells at 24 h (Fig. 3B, uptake), which was 60- and 88-fold higher than CAB LAP (2.4 nmoles/10^6^) and NCAB (1.6 nmoles/10^6^ cells), respectively. In retention study (Fig. 3B, retention), NMCAB was retained within the cells for up to 30 days, however CAB levels were not detectable in NCAB and CAB LAP treated cells after 1 or 5 days. Both MCAB and CAB were detected in NMCAB treated cells indicating that MCAB is hydrolyzed inside cells to release the CAB. To investigate this proposed mechanism, we used TEM to visualize retained nanoparticles. We observed crystalline structures inside cell compartments of NMCAB treated MDM after 8 h of drug loading, but importantly not in replicate cells treated with CAB LAP (Fig. 3C, uptake). After a 48-h washout period, crystals remained in NMCAB treated MDM, confirming that NMCAB crystals remain stable in cytoplasmic organelles for extended time periods.

### 3.4. Cell-based antiretroviral activity

**Fig. 4:**
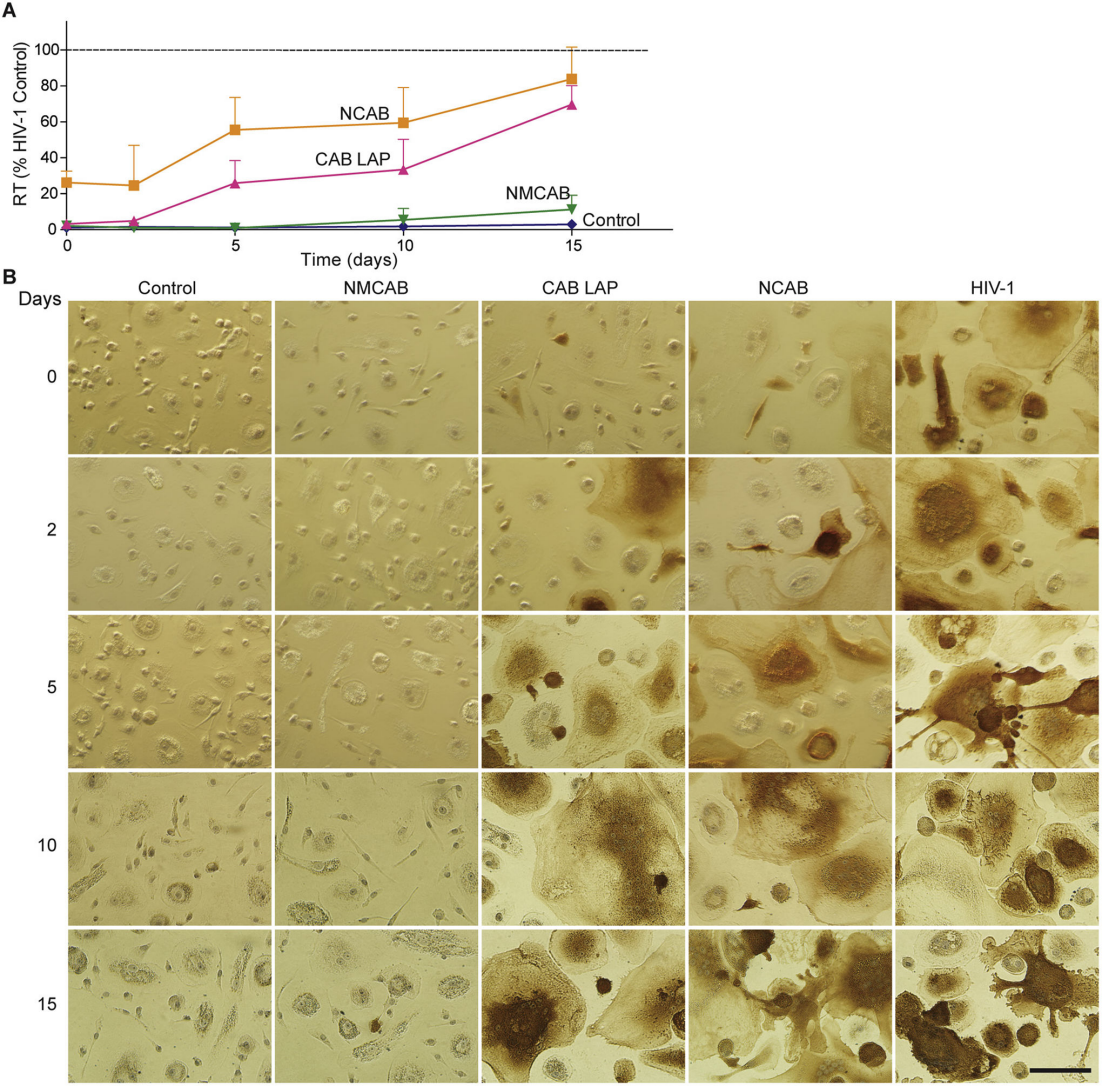
NMCAB protects MDM from HIV-1 infection over time MDM were treated with NMCAB, CAB LAP or NCAB containing 100 µM drug for 8h. At days 0, 2, 5,10, and 15 post drug loading, MDM were challenged with HIV-1_ADA_ at 0.1 MOI for 4 h. Uninfected cells without treatment served as negative controls (Control); HIV-1 infected cells without any treatment served as positive controls (HIV-1). Samples were collected for antiretroviral activity test seven days after viral challenge. (**A**) HIV RT activities were measured in culture media. Results are shown as percentage of RT activities as compared to HIV-1 infected MDM. Data are expressed as mean ± SD for *n* = 6 samples per group. (**B**) Cells were fixed in paraformaldehyde and stained for HIV-1p24 antigen (brown). Scale bar: 100 µm. (For interpretation of the references to colour in this figure legend, the reader is referred to the web version of this article.)

To assess whether increased uptake and retention of NMCAB translate into improved antiretroviral efficacy, MDM were treated with NMCAB, NCAB, or CAB LAP for 8 h, followed by HIV-1_ADA_ challenge (MOI of 0.1 infectious viral particles/cell) at days 0, 2, 5, 10, and 15 (Fig. 4). Enhanced antiretroviral activity was observed in cells treated with NMCAB compared to CAB LAP or NCAB. Media HIV RT activity in NMCAB treated cells was significantly lower than that of CAB LAP and NCAB treated cells (Fig. 4A). For CAB LAP or NCAB treated cells, RT activity was suppressed only within the first two days of treatment, followed by increases to 70 and 84% of the HIV-1 infected control at day 15, respectively. In contrast, sustained viral inhibition was observed in NMCAB treated cells for up to 15 days. These findings were confirmed by HIV-1p24 staining, where no brown positive cells were detected up to 10 days, with rare infected cells seen at day 15 in NMCAB treated MDM. In contrast, for both CAB LAP and NCAB treatments HIV-1p24 positive cells were readily observed at day 0 and increased to a level that is similar to HIV-1 positive control at subsequent time points (Fig. 4B). These data paralleled the rapid washout of CAB LAP and NCAB from the cells compared to sustained NMCAB crystals.

### 3.5. PK and BD assessments

**Fig. 5:**
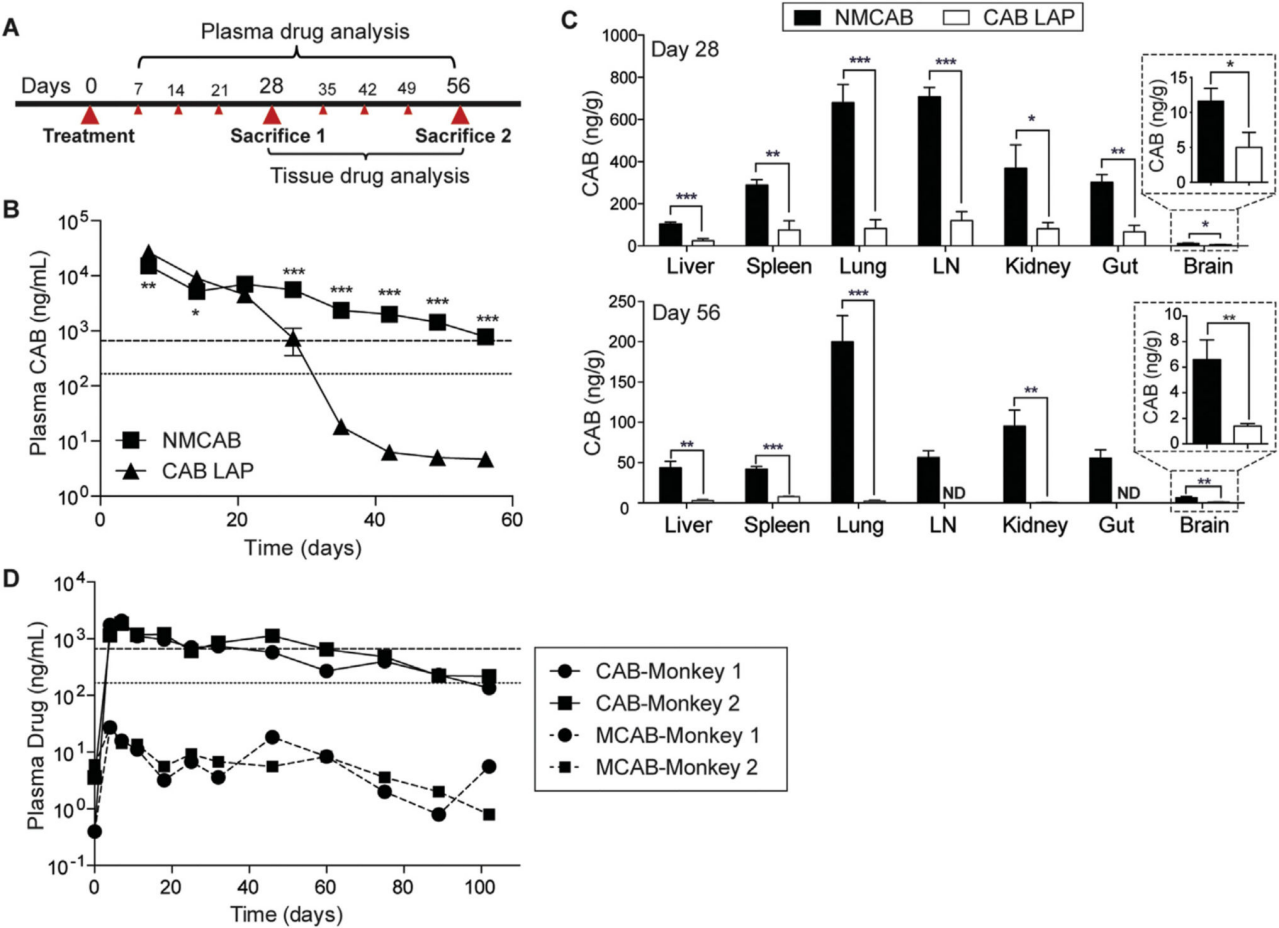
NMCAB PK and BD profiles (**A**) Experimental timeline. BALB/cJ mice were administered intramuscularly with 45 mg/kg CAB equivalent of CAB LAP or NMCAB at day 0 followed by weekly blood collection to day 56. Mice were sacrificed at day 28 and 56 respectively. (**B**) Plasmas were isolated at each blood collection, and then analyzed for CAB concentrations by UPLC/MS/MS. Horizontal dotted and dashed lines represent 1 × and 4 × PA-IC_90_, respectively. Data represent mean ± SEM for *n*= 5 mice per group and were compared by multiple *t*-test. *P < 0.05, **P < 0.01, ***P < 0.001. (**C**) Tissues were collected at each sacrifice day and then analyzed for CAB concentrations by UPLC/MS/MS. LN: lymph node. Data are expressed as mean ± SEM for *n*= 5 mice per group. *P <0.05, **P <0.01, ***P <0.001 by Student’s *t*-test (**D**) Two Chinese rhesus macaques were administered intramuscularly with 45 mg CAB equivalents/kg NMCAB. Plasma CAB and MCAB concentrations were monitored up to 102 days.

Male BALB/cJ mice were intramuscularly administered 45 mg CAB equivalents/kg of NMCAB or CAB LAP to assess PK and BD profiles for 8 weeks, the study timeline is shown in Fig. 5A. NMCAB treatment showed lower initial concentrations but a slower CAB decay rate compared to CAB LAP treatment. Compared to CAB LAP treated mice, significantly higher plasma CAB concentrations were achieved in NMCAB treated mice starting from day 21. Single injection of NMCAB sustained the CAB plasma drug concentrations above 4 × PA-IC_90_ [24] for up to two months. At day 56, average plasma CAB concentration was 780 ng/mL in NMCAB treated mice. In contrast, plasma CAB concentrations in mice treated with CAB LAP fell below 4 × PA-IC_90_ by day 35 (Fig. 5B). In a PK study with a lower dose of 15 mg CAB equivalents/kg (Fig. S2), NMCAB treatment maintained plasma CAB concentrations above 4 × PA-IC_90_ for 28 days, which equals what CAB LAP treatment could achieve with a 45 mg/kg dose. PK parameters were calculated for all drug formulations at a 45 mg/kg dose (Table 1). The apparent terminal phase half-life (t_1/2_) after NMCAB intramuscular administration was 4-fold greater than for CAB LAP (278 compared to 71 h). Similarly, CAB mean residence time (MRT) was 2-fold longer for NMCAB compared to CAB LAP. The extended t_1/2_ of NMCAB was the result of a 4-fold increase in volume of distribution (Vβ/F), whereas clearance (CL/F) was comparable between all tested formulations. Up to 95-fold increases in CAB concentrations were detected in liver, lungs, spleen and lymph nodes in NMCAB treated animals at both days 28 and 56 (Fig. 5C). Assuming tissue density is 1 g/mL, NMCAB treatment provided tissue CAB concentrations above PA-IC_90_ (166 ng/mL) for all the tissues including lung, lymph node, kidney, gut and spleen, except for liver and brain, 28 days after initial injection. In contrast, for CAB LAP treated mice, drug concentrations were below PA-IC_90_ in all of the tested tissues. At day 56, tissue drug levels in CAB LAP treated mice were close to or below the detection limit. In contrast, significantly higher drug levels in NMCAB treated mice were detected. Notably, CAB concentration was still above PA-IC_90_ in lungs.

A preliminary NMCAB PK study in non-human primates has supported our findings in rodents. Plasma CAB concentrations were at or above PA-IC_90_ for up to 102 days in two male rhesus macaques given a single intramuscular injection of NMCAB at 45 mg CAB equivalents/kg (Fig. 5D). Plasma CAB concentrations reached a maximum at day 7 and were maintained from day 11 through day 102. This is in contrast to previous studies on the PK of CAB LAP in male rhesus macaques [19], which showed that 10 weeks after a 50 mg/kg intramuscular dose, plasma CAB concentrations were below the PA-IC_90_ for all the animals with rapid decay. Calculated PK parameters (Table S2) demonstrated a prolonged t_1/2_ of 22.5 and 27.2 days, respectively for each monkey, which is approximately 2–4 fold as compared to previous reported t_1/2_ (5–12 days) for CAB LAP in male macaques [19]. Complete blood counts (CBC) and liver and kidney metabolic profiles were unchanged before and after NMCAB treatment (Table S3).

### 3.6. NMCAB depot analyses

**Fig. 6:**
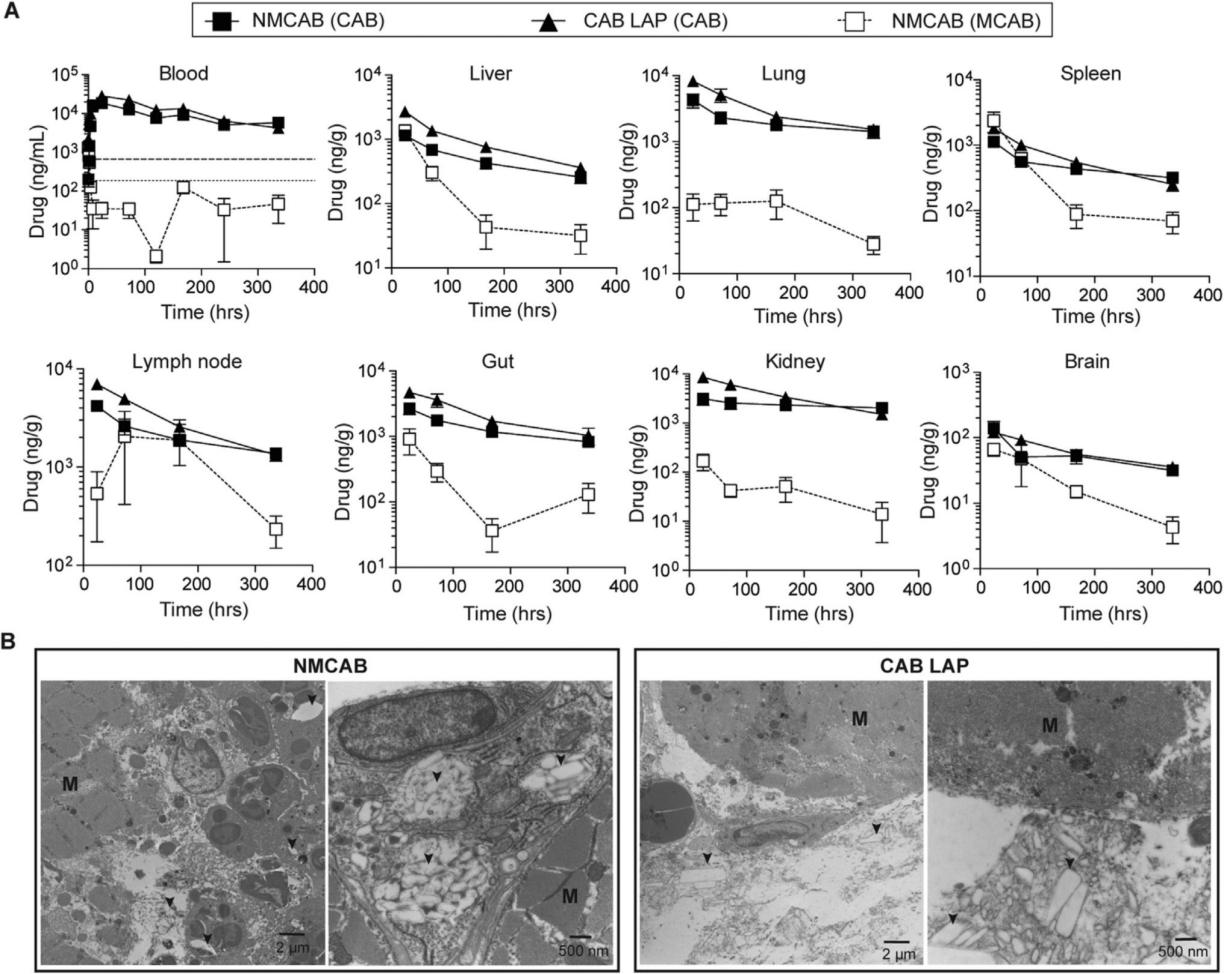
Drug depot analyses in BALB/cJ mice (**A**) Tissue drug depot analysis. Time course of whole blood and tissue drug levels in BALB/cJ mice receiving a single intramuscular injection of NMCAB or CAB LAP at 45 mg CAB equivalents/kg for early time points up to 14 days. Both CAB (solid lines) and MCAB (dashed lines) were measured in NMCAB treated mice. Horizontal dotted and dashed lines represent 1 × and 4 × PA-IC_90_, respectively. Data are expressed as mean ± SEM for *n*= 5 mice per group. (**B**) Injection site depot analysis. BALB/cJ mice receiving intramuscular injection of CAB LAP or NMCAB were sacrificed 24 h after drug administration. Injection site muscles were collected and analyzed by TEM. Representative figures of muscle cross-sections are shown. The muscle bundles are depicted as “M”; arrows point drug crystals.

Unlike the high CAB concentrations at later time points, NMCAB treatment resulted in lower initial plasma CAB concentration as compared to CAB LAP. For example, day 7 after 45 mg/kg drug administration, CAB plasma concentrations were 15,185 ng/mL in NMCAB treated mice versus 26,678 ng/mL in CAB LAP treated mice (*P* = 0.0089) (Fig. 5B). This suggests a secondary depot and/or a slower release of the drug from injection depot. Therefore, a PK and BD study focusing on initial distribution of both MCAB and CAB was conducted to identify the potential tissue depot for the NMCAB formulation. Blood MCAB concentrations in NMCAB treated mice were 1300 ng/mL at 15 min after an intramuscular injection of 45 mg CAB equivalents/kg but dropped below 200 ng/mL at 2 h (Fig. 6A). These results suggested that the MCAB was readily cleaved in blood. Twenty-four hrs after injection, blood MCAB concentration was only 35 ng/mL in NMCAB treated mice, however in tissues, MCAB concentrations were higher than the blood MCAB concentration. Notably, spleen (2369 ng/g), liver (1358 ng/g) and gut (908 ng/g) exhibited highest MCAB concentrations at 24 h (Fig. 6A). Lymph nodes exhibited a slower distribution, the highest MCAB concentration (2057 ng/g) was observed at 72 h after injection. Given the fact that NMCAB more readily penetrates cells (Fig. 3B and C), the high MCAB concentrations in tissues versus negligible concentrations in blood suggest that tissues, such as spleen, lymph node, liver and gut, can serve as drug depots for NMCAB formulation.

Injection site depots were also investigated. TEM analysis (Fig. 6B) revealed robust immune cell infiltration within the muscle at the site of injection in NMCAB treated mice. The immune cells, including neutrophils and macrophages, were inside the muscle bundles (M) carrying large amount of drug crystals (arrows) in the cytoplasm. However, in CAB LAP treated mice, only a few immune cells were recruited to the muscle. In addition, these immune cells were not actively taking up the drug crystals, as indicated by the majority of the drug crystals being observed between the muscle bundles and outside the immune cells.

### 3.7. Studies of relative viral restriction for NMCAB and CAB LAP

**Fig. 7:**
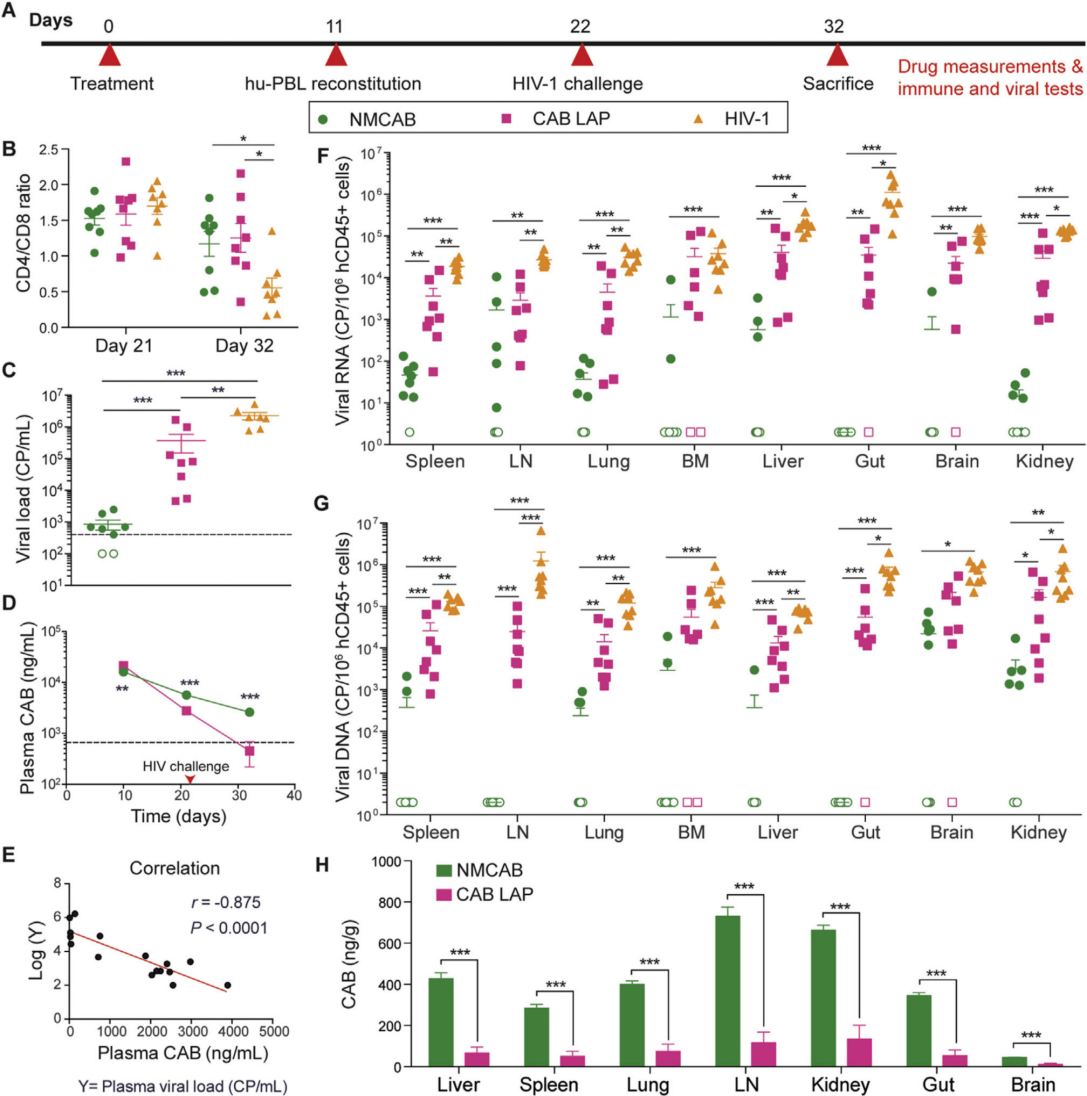
Viral restriction of NMCAB compared to CAB LAP in hu-PBL reconstituted NSG mice (**A**) Experimental timeline. NSG mice were treated intramuscularly with NMCAB or CAB LAP (45 mg CAB equivalents/kg) at day 0, followed by hu-PBL reconstitution at day 11, HIV-1_ADA_ challenge at day 22, then sacrifice at day 32. (**B**) Peripheral blood CD4/CD8 T-cell ratios at days 21 and 32 were analyzed by flow cytometry. Data represent mean ± SEM (*n*= 8) and were compared by one-way ANOVA followed by Holm-Sidak post hoc test. (**C**) Plasma viral loads were measured at day 32. Open symbols represent samples that were below the detection limit (400 copies/mL). Data represent mean ± SEM (*n*= 8) and were compared by one-way ANOVA followed by Dunnett T3 post hoc test (**D**) Plasma CAB concentrations were detected on days 10,21, and 32. Horizontal dashed line represents 4 × PA-IC_90_. Data represent mean ± SEM (*n*= 8) and were compared by multiple *t*-test. (**E**) Correlation between plasma CAB concentrations and log_10_ plasma viral load on day 32 in drug treated mice was computed. Pearson correlation coefficient (*r*) = −0.875, *P* < 0.0001. (**F**) Viral RNA and (**G**) DNA in tissues were detected by semi-nested real time PCR. Values are presented as nucleic acid viral copies per 10^6^ human CD45^+^ cells. LN: lymph node; BM: bone marrow. Open symbols represent samples that were below the detection limit Data represent mean ± SEM (*n*= 8) and were compared by one-way ANOVA followed by Dunnett T3 post hoc test. (**H**) CAB concentrations in tissues were detected in NMCAB or CAB LAP treated mice on day 32. Data represent mean ± SEM (*n*= 8) and were compared by Student’s *t*-test. *P <0.05, **P <0.01, ***P < 0.001.

To determine whether the improved PK profile of NMCAB could translate into improved antiretroviral efficacy, NSG mice were administered a single intramuscular injection of NMCAB or CAB LAP at 45 mg CAB equivalents/kg on day 0, followed by human PBL reconstitution on day 11, challenge with HIV-1_ADA_ on day 22, and necropsy 10 days after HIV-1 challenge (Fig. 7A). Blood, plasma, and tissues were collected to determine drug levels, CD4/CD8 ratio, HIV-1p24 expression, viral load, and viral RNA/DNA. Flow cytometry analysis showed that both NMCAB and CAB LAP treatment protected the animals from HIV-1-induced CD4^+^ Tcell loss (Fig. 7B). Plasma viral load test demonstrated that NMCAB treatment provided superior viral restriction compared to CAB LAP treatment. Plasma viral load was not detectable in 2/8 animals in the NMCAB treatment group. The average viral load in the remaining 6 animals was 1.1 × 10^3^ copies/mL as compared to 2.3 × 10^6^ copies/mL in untreated HIV-1 controls and 3.7 × 10^5^ copies/mL in CAB LAP pre-treated animals, respectively (Fig. 7C). Plasma CAB concentrations were above the 4 × PA-IC_90_ throughout the entire study in the NMCAB treatment group, whereas CAB LAP treatment failed to maintain plasma CAB concentrations above 4 × PA-IC_90_ by the end of the study (Fig. 7D). Pearson correlation analysis revealed a strong negative correlation between plasma CAB concentration and log_10_ plasma viral load (Fig. 7E) on day 32, highlighting the importance of maintaining sufficient drug concentrations to achieve ideal viral suppression. Immunohistochemical analysis revealed that 7/8 animals in the NMCAB treated group were HIV-1p24 negative in spleen and lung, while HIV-1p24 positive cells were observed in all the CAB LAP treated mice and untreated HIV-1 control animals. Representative images of HLA-DR and HIV-1p24 staining are shown in Fig. S3. In all tissues, including spleen, lung, lymph node, and bone marrow, NMCAB treatment reduced viral RNA and DNA levels by over an order of magnitude, and in many tissues, levels were reduced over 3 logs_10_ compared to HIV-1 positive controls or CAB LAP treated CAB LAP treated mice (Fig. 7F and G). Notably, out of the 8 mice in NMCAB treatment group, viral RNA was below the limit of detection in 1 animal for spleen, 3 for lymph node, 3 for lung, 6 for bone marrow, 5 for liver, 8 for gut, 7 for brain, and 4 for kidney. Similar trend was observed in viral DNA as well. The reduced tissue viral RNA, DNA and HIV-1p24 expression correlated with > 5-fold increases in CAB levels in tissues (Fig. 7H).

## 4. Discussion

The era of long-acting ARVs has now emerged [28,44]. CAB and RPV can be used at dosing intervals for up to every two months in HIV-1 infected patients and serve to keep viral replication at check [14]. However, with more than 20 approved ARVs in North America and Europe, whether the broad therapeutic choices of conventional daily dosing [45] can be translated into less frequent administrations have not yet been realized. Broader dosing intervals are of real potential benefit with current ARV treatments as, when initiated, patient adherence to the offered regimen is imperative, otherwise resistance patterns will rapidly emerge. Unfortunately, for a broad range of reasons that include access limitations, stigma, cost, substance abuse, absorption abnormalities, and comorbidities, patients often do not adhere to regimen recommendations [8,11,46,47]. While LAP therapeutics will soon become available, they most likely will have a specific niche due, in part, to limitations associated with large injection volumes, injection site reactions, and suboptimal dosing intervals [25,48]. Apparent means to overcome these limitations rest with decreasing required injection volumes, broadening of dosing intervals, and targeting viral reservoirs. This is where we believe nanotechnologies can occupy this important niche.

In particular, the current report addresses such inherent limitations through creation of a CAB prodrug that can be readily formulated into nanoparticles. The possibility for achieving enhanced viral reservoir entry, retention and long-term efficient release was recently demonstrated for abacavir (ABC) and lamivudine (3TC) [35,49]. MCAB, like modifications in ABC and 3TC, was designed to be cleaved in the presence of esterases, and to release native drug as well as myristic acid. Notably, myristic acid also has antiretroviral activity through inhibition of *N*-myristoyltransferase, an enzyme that affects the myristoylation of the viral life cycle related proteins [50–53].

Amphiphilic P407 surfactant was used to stabilize NMCAB nanosuspensions. Lipophilic MCAB interacts with the hydrophobic core of the polymer to produce homogeneous surfactant coated nanocrystals. The formulation process of NMCAB was optimized to maximize drug loading with limited excipient usage, while maintaining scalability and long-term storage stability. This could potentially translate into reduced dosing volumes and decreased excipient-related adverse effects. In fact, a significantly higher reduction (*P* <0.001) in MDM cell viability was observed upon treatment with 400 µM CAB equivalents of CAB LAP as compared to NMCAB (Fig. 3A). We believe this is due to the higher amount of excipients, especially surfactant, in the CAB LAP formulation [16]. NMCAB formulation has comparable particle size, polydispersity, and morphology as CAB LAP formulation. Previous studies have shown that particle morphology plays an important role in nanoparticle attachment and internalization by macrophages [42,43]. Both NMCAB and CAB LAP exhibited rod shape morphology (Fig. 2C), the preferred geometry for macrophage uptake.

CAB is a hydrophobic compound with a sustained release profile now administered as a single-dose LAP. Monthly to bimonthly dosing is readily achievable due to the drug’s extended half-life, high protein binding capacity, and slow dissolution rate [16,24,54]. The long half-life of CAB after intramuscular injection is mostly due to the slow release of the drug from injection site depots. This is notable and observed in animal studies at muscle site depots. In contrast, such drug depots were not observed in replicate animals injected with NMCAB. Instead, abundant numbers of immune cells, including macrophages and neutrophils, appeared at the site of injection carrying drug crystals in cytoplasmic organelles after injection. These findings suggested a fast cell entry and redistribution of the NMCAB nanoparticles. After cell entry, NMCAB undergoes slow dissolution to release the prodrug that is then hydrolyzed back into active CAB. The change of major drug depot from muscle injection site to cells and tissues may also reduce injection site reactions. It is important to note that the myristic acid lipophile on MCAB facilitates cell penetration and enhanced long term nanocrystal stability.

High concentrations of MCAB were observed in spleen, liver, gut and lymph node shortly after nanoformulation administration despite negligible MCAB concentrations in blood (Fig. 5A) suggesting that reticuloendothelial system plays a critical role in NMCAB redistribution. These data sets parallel previous findings from our laboratory that monocyte-macrophages are readily harnessed for ARV delivery based on high loading capacities and ability to carry cargoes across physiological barriers [55,56]. Notably, they are also major reservoir sites for HIV-1 adding to the potential improvements in antiretroviral responses observed in this report.

The mechanisms for extended half-life of NMCAB rests beyond its structural properties and includes reduced drug metabolism. This was achieved by blocking of its hydroxyl group through pro-drug esterification. Altogether, in mice NMCAB treatment provided a 4-fold longer half-life than CAB LAP with reduced peak concentrations, reflecting a slower release of the drug from depots [17]. With lower carboxylesterase activity and a slower metabolic rate in humans compared to mice [57,58], NMCAB could extend dosing intervals beyond two months and reduce overall dosage and injection volumes. These were, in part, supported by our own PK determinations of NMCAB in rhesus macaques.

CAB LAP has been demonstrated to be highly protective against viral challenge in non-human primates irrespective of route of infection [19–22]. In our comparisons of viral restriction we used human PBL-reconstituted NSG mice as a screening tool. While plasma CAB concentrations were above 4 × PA-IC_90_ throughout the study, HIV infection was, nonetheless, still observed. We posit that this was due to several aspects of this mouse model. *First* is xenoreactivity. In human PBL-reconstituted mice, engraftment of mature human lymphocytes commonly leads to graft-versus-host effects. This enables human lymphocyte activation and an increased susceptibility to HIV-1 infection [59]. *Second* is a high rate of available viral target cells. Human CD4^+^ T cell percentage after reconstitution was 52% of total CD3^+^ cells providing a large pool of host cells. *Third* is the high dose of administered HIV-1. 10^4^ TCID_50_ was used to infect PBL-reconstituted mice. Under these circumstances, CAB LAP could not achieve protection against HIV challenge as it has achieved in rhesus macaques [19–22]. Nonetheless, the enhanced susceptibility to HIV challenge in this model provided a “proof of concept tool” to compare between NMCAB and CAB LAP. It is noteworthy that NMCAB treated animals showed significantly lower plasma and tissue viral loads as compared to CAB LAP treated mice. These improvements are based on its ability to maintain plasma drug levels above 4 × PA-IC_90_ for a prolonged time beyond what CAB LAP can provide. Future studies using more comprehensive humanized mouse model and SHIV challenge in rhesus macaques will provide more information on NMCAB’s ability to prevent new infections.

Altogether, while HIV-1 infection is treatable, the requirements of daily or more frequent ARV dosing preclude achieving effective drug concentrations and preventing viral resistance patterns. While long-acting ARVs such as CAB LAP and RPV LAP can be administered monthly to bimonthly and improve regimen adherence, its widespread use would be better realized if the dosing interval could be extended further with reduced injection site reactions. This study provides possible solutions to such technical hurdles, and as such, impacts the broader use of long-acting ARVs. This was made possible by chemical modification and nanoformulation strategies though their abilities to affect drug cellular entry and depot formation. The secondary depot beyond injection site could not only reduce administration frequency, but also serve to better extend viral restriction in HIV residing tissues, including lymph nodes, gut-associated lymphoid tissues and spleen [60–62]. Moving forward, NMCAB could be further optimized by additional chemical modifications, surface decorations for cell targeting and combination with other ARVs in a single formulation. These strategies will further extend dosing intervals, ease administrations, and facilitate drug carriage across physiological barriers to better reach virus-susceptible cells to improve clinical applications.

## 5. Conclusions

In summary, NMCAB, a nanoformulated prodrug of CAB, has been successfully developed with appreciable stability and scalability. It exhibits enhanced cellular entry and retention, and forms intracellular drug depots that provide sustained and effective drug release and long-term protection against HIV challenge in macrophages. Rodent or rhesus macaque PK, distribution, and relative viral restriction evaluations revealed that NMCAB is able to extend dosing intervals, enhance tissue drug concentrations, and improve antiretroviral profiles beyond the current CAB LAP formulation. We believe these improvements will positively affect outcomes for both HIV treatment and prevention.

## Supplementary Material

supplemental files

## Figures and Tables

**Table 1:** BALB/cJ mice noncompartmental PK analysis.

Parameters	Treatment	
	
	CAB LAP	NMCAB
λ_Z_ (1/h)	0.00974 ± 0.00017	0.0025 ±0.00005
t_1/2_(h)	71.3 ± 1.2	277.8 ± 5.9
AUC_last_ (h ng/mL)	6883997.7 ± 511013.4	6584058.1 ± 247768.7
AUC_0-∞_ (h ng/mL)	6884479.4 ± 511007.1	6897132.5 ± 260736.5
AUC % Extrapolation	0.01 ± 0.00	4.53 ± 0.35
V_β/F_ (L/kg)	0.69 ± 0.06	2.64 ± 0.13

Data are expressed as mean ± SEM (*n* = 5).
