# Supplementary material for: Creation of a nanoformulated cabotegravir prodrug with improved antiretroviral profiles
Source: Biomaterials. Author manuscript; Available in PMC 2018 Apr 30. (PMC5926202; doi:10.1016/j.biomaterials.2017.10.023)
Supplement: supplemental files [file NIHMS960805-supplement-supplemental_files.pdf]

Supplemental materials

Supplemental figures

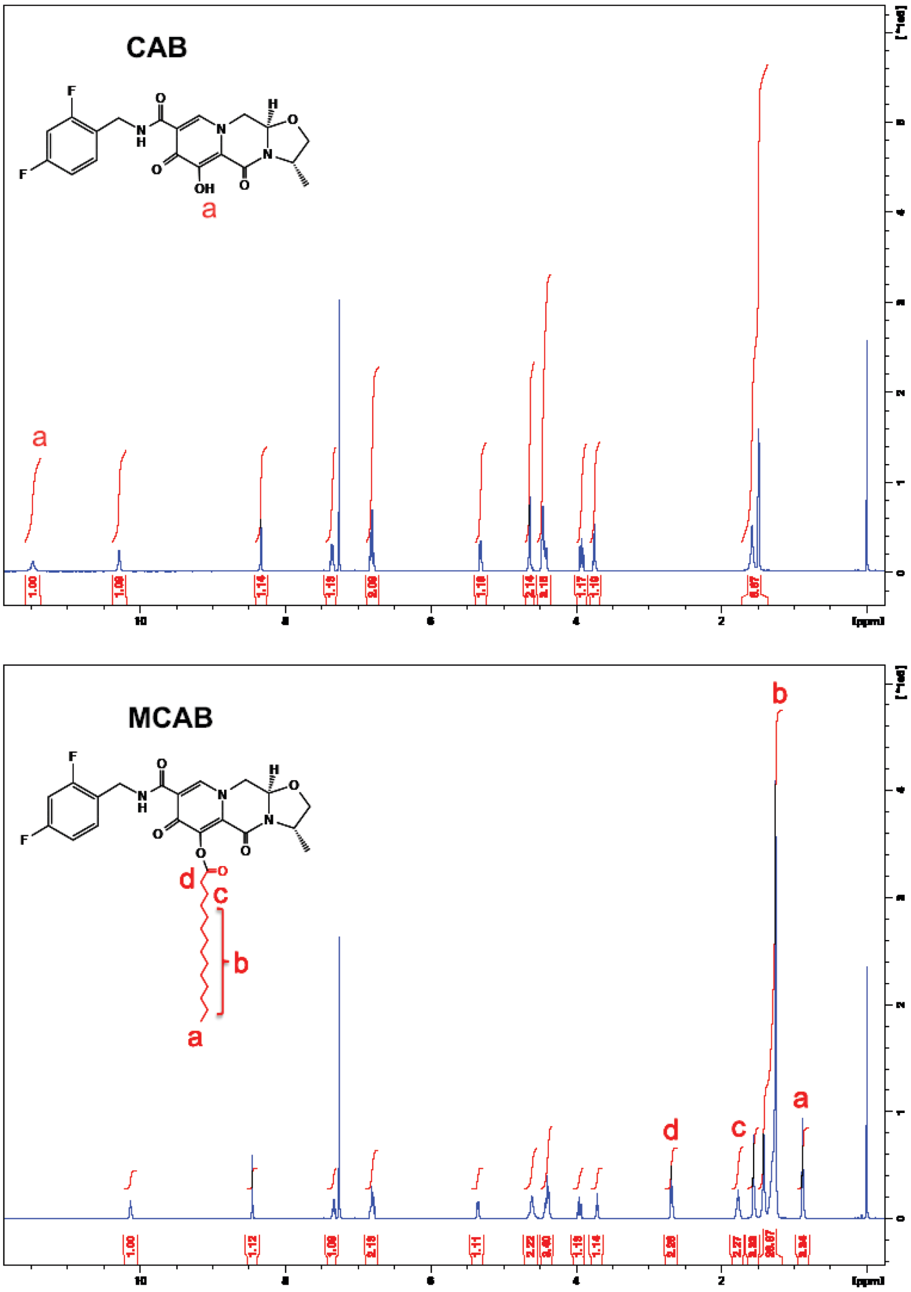

Figure S1. ^{1}H-NMR spectra of CAB and MCAB. Triplets at 0.88, 1.77 and 2.69 ppm and a broad singlet at 1.26 ppm correspond to protons obtained from the derivatizing MCAB MCAB fatty acid alkyl chain. A phenol proton peak at 11.5 ppm was observed in the CAB spectra but not for MCAB. This confirms the substitution of the CAB hydroxyl proton with myristic acid.

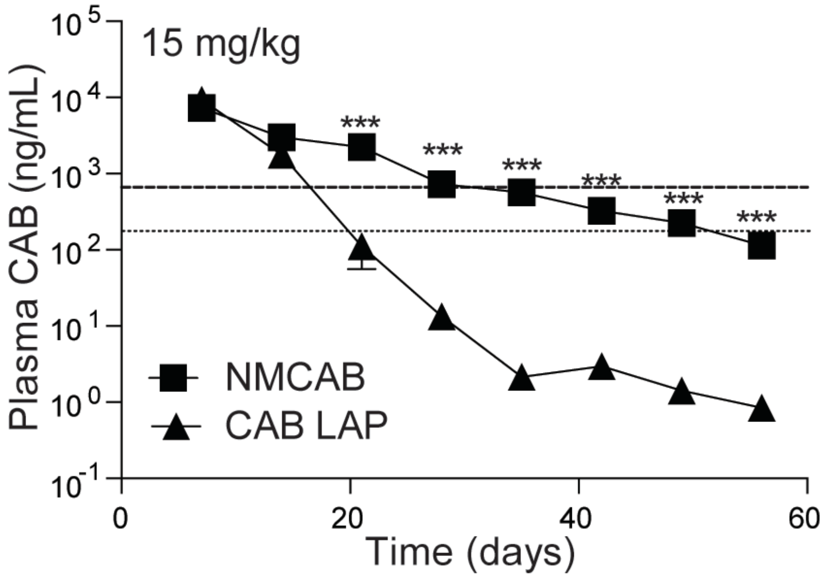

Figure S2. NMCAB PK in BALB/cJ mice with low dose of 15 mg CAB equivalents/kg dose. BALB/cJ mice were administered intramuscularly with 15 mg/kg CAB equivalent of CAB LAP or NMCAB at day 0 followed by weekly blood collection to day 56. Data represent mean ± SEM for *n* = 5 mice per group and were compared by multiple t test. *** $P < 0.001$.

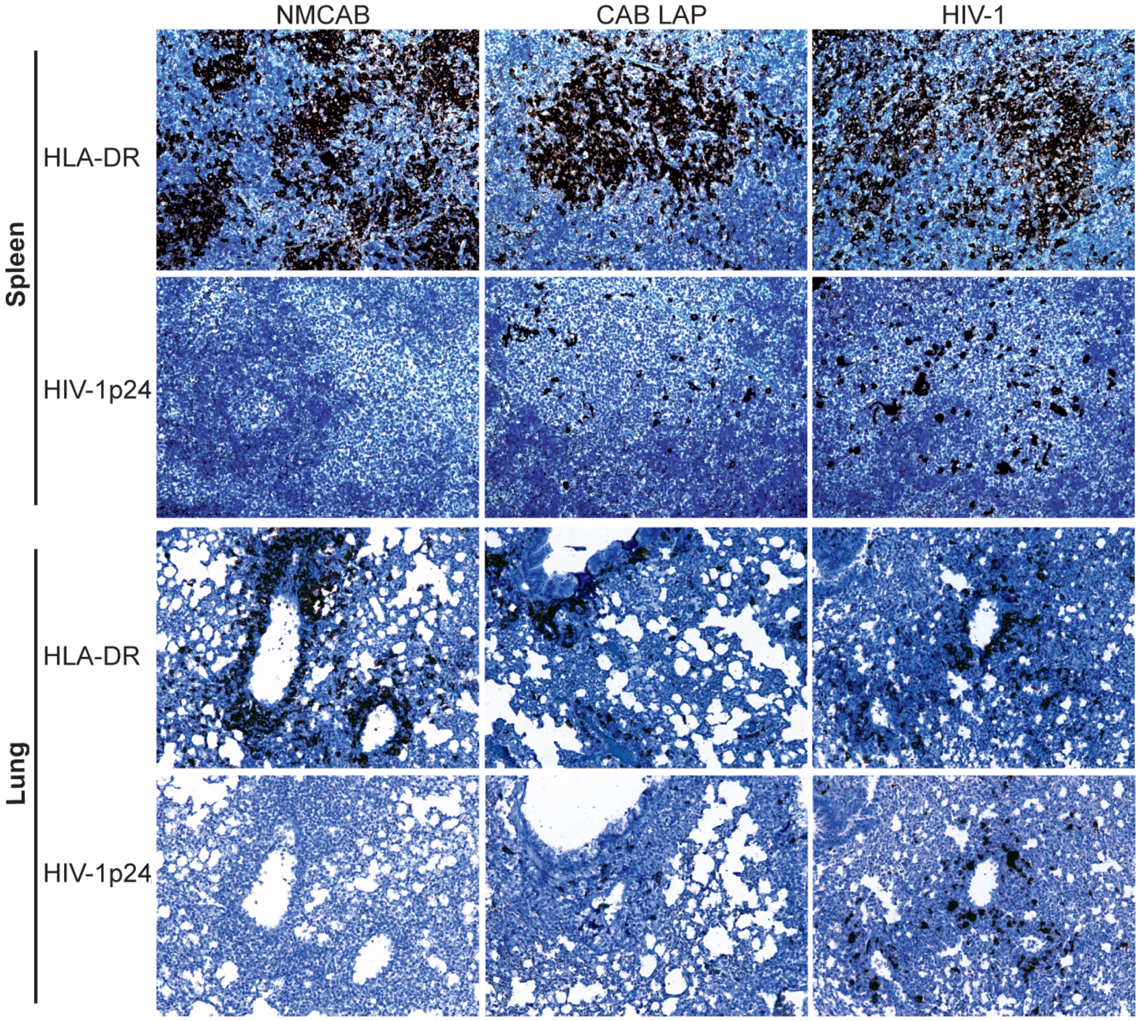

Figure S3. Representative HIV-1p24 staining for viral restriction study. NSG mice were treated intramuscularly with NMCAB or CAB LAP (45 mg CAB equivalents/kg) at day 0, followed by hu-PBL reconstitution at day 11, HIV-1_{ADA} challenge at day 22, then sacrifice at day 32. Levels of HLA-DR and HIV-1p24 antigen in spleen and lung of human PBL-reconstituted NSG mice treated with NMCAB, CAB LAP, and HIV-1 control mice 10 days after HIV-1 challenge.

Supplemental tables

Table S1. Physicochemical properties of NMCAB, MCAB, and CAB LAP

Formulation	D_{eff} (nm)[a]	PdI[a]	ζ-potential (mV)[a]
**NMCAB**	318 ± 25	0.21 ± 0.02	-22.0 ± 3.4
**NCAB**	315 ± 26	0.28 ± 0.04	-8.2 ± 1.4
**CAB LAP**	257 ± 6	0.22 ± 0.02	-23.2 ± 1.7

[a]Effective diameter (D_{eff}), polydispersity index (PdI) and ζ-potential were determined in water. Data are expressed as mean ± SD (*n* = 3).

Table S2. Rhesus macaque non-compartmental PK analysis

PK Parameters	Monkey 1	Monkey 2	Average
**λ_Z (1/day)**	0.0254	0.0308	**0.0281**
**$t_{1/2}$ (day)**	27.2	22.5	**24.9**
**AUC_{last} (day*ng/mL)**	60661.6	74987.4	**67824.5**
**AUC0-∞ (day*ng/mL)**	65959.9	82096.9	**74028.2**
**AUC % Extrapulation**	8.0	8.7	**8.3**
**$V_{\beta/F}$ (L/kg)**	26.8	17.8	**22.3**
**CL/F (L/day/kg)**	0.68	0.55	**0.62**
**MRT 0-∞ (days)**	42.3	46.9	**44.6**

Table S3. Complete blood counts and metabolic panel of rhesus macaques administrated with NMCAB

**Monkey 1**				**Days**				
**Parameters**	**0**	**4**	**7**	**11**	**18**	**25**	**32**	**46**
WBC Count ($\times10^6/\mu L$)	10.8	10	10.7	11.7	8.6	11.1	11	10
RBC Count ($\times10^6/\mu L$)	4.97	4.72	4.64	4.54	4.48	4.79	4.91	4.93
Hemoglobin (g/dL)	12.1	11.5	11.2	11	10.9	11.7	11.9	12
Hematocrit (%)	36.5	36.1	33.3	34.3	33.3	36.3	37.3	37.8
MCV (fL)	73.4	76.5	71.8	75.6	74.3	75.8	76	76.7
MCHC (%)	33.2	31.9	33.6	32.1	32.7	32.2	31.9	31.7
RBC Distrib Width (%)	15.2	14.6	15.1	15.5	16.1	15.4	15.7	14.8
Platelet Count ($\times10^3/\mu L$)	363	374	448	446	351	374	397	353
Neutrophil Seg (%)	8	6	18	10	10	9	18	15
Neutrophil Band (%)	0	0	0	1	0	0	0	0
Lymphocyte (%)	80	91	80	85	84	81	78	84
Monocyte (%)	11	2	2	3	5	9	3	0
Eosinophil (%)	1	1	0	1	1	1	1	1
Basophil (%)	0	0	0	0	0	0	0	0
Nucleated RBC (/diff)	0	0	0	0	0	0	0	0
Absolute Neutrophil ($\times10^3/\mu L$)	0.9	0.6	1.9	1.3	0.9	1	2	1.5
Absolute Lymphocyte ($\times10^3/\mu L$)	8.6	9.1	8.6	9.9	7.2	9	8.6	8.4
Absolute Monocyte ($\times10^3/\mu L$)	1.2	0.2	0.2	0.4	0.4	1	0.3	0
Absolute EOS ($\times10^3/\mu L$)	0.1	0.1	0	0.1	0.1	0.1	0.1	0.1
Absolute BASO ($\times10^3/\mu L$)	0	0	0	0	0	0	0	0
AST (U/L)	25	50	32	31	31	27	32	28
Alkaline Phosphatase (U/L)	810	624	654	584	639	668	637	674
Bilirubin Total (mg/dL)	0.1	0.1	0.1	0.1	0.1	0.1	0.2	0.1
Calcium (mg/dL)	10.2	9.7	10	10	10	10	10.2	10.3
Protein Total (g/dL)	6.8	6.4	6.4	6.5	3.1	6.7	6.8	6.6
Albumin (g/dL)	4.5	4	4.1	4.3	4.3	4.5	4.7	4.5
Glucose/Random (mg/dL)	101	92	74	77	52	77	82	69
Urea Nitrogen (mg/dL)	21	23	20	22	21	21	22	21
Creatinine (mg/dL)	0.56	0.54	0.5	0.51	0.53	0.49	0.55	0.54
Bun/ Creatinine Ratio (mgUN/mgCR)	37.5	42.6	40	43.1	39.6	42.9	40	38.9
Sodium (mmol/L)	145	145	144	144	146	144	146	146
Potassium (mmol/L)	3.2	3.5	3.8	3.6	3.3	3.7	3.3	3.8
Chloride (mmol/L)	107	104	106	105	105	106	106	108
Osmolality / Calc (mOsm/kg)	302	302	298	299	301	298	303	302
Carbon Dioxide (mmol/L)	16	18	23	23	24	21	19	23
Anion Gap (mmol/L)	22	23	15	16	17	17	21	15
ALT (U/L)	53	90	78	60	35	37	36	47

Table S3 continued

**Monkey 2**				**Days**				
**Parameters**	**0**	**4**	**7**	**11**	**18**	**25**	**32**	**46**
WBC Count (×10⁶/µL)	3.4	6	3.4	5.7	5.5	4	3.8	4.5
RBC Count (×10⁶/µL)	4.92	4.92	4.69	4.57	4.68	4.6	4.56	5.02
Hemoglobin (g/dL)	11.8	11.8	11.3	11	11.4	11	10.8	12
Hematocrit (%)	35.8	37.8	33.5	34.5	34.8	34.8	35	38.6
MCV (fL)	72.8	76.8	71.4	75.5	74.4	75.7	76.8	76.6
MCHC (%)	33	31.2	33.7	31.9	32.8	31.6	30.9	31.1
RBC Distrib Width (%)	12	11.9	12.3	12.4	12.7	12.4	12.7	12.3
Platelet Count (×10³/µL)	342	358	372	401	372	330	367	326
Neutrophil Seg (%)	26	35	32	32	48	31	24	30
Neutrophil Band (%)	0	0	0	1	0	0	0	1
Lymphocyte (%)	72	60	62	57	47	63	68	56
Monocyte (%)	2	3	5	5	0	5	8	3
Eosinophil (%)	0	2	1	5	5	1	0	10
Basophil (%)	0	0	0	0	0	0	0	0
Nucleated RBC (/diff)	0	0	0	0	0	0	0	0
Absolute Neutrophil (×10³/µL)	0.9	2.1	1.1	1.9	2.6	1.2	0.9	1.4
Absolute Lymphocyte (×10³/µL)	2.4	3.6	2.1	3.2	2.6	2.5	2.6	2.5
Absolute Monocyte (×10³/µL)	0.1	0.2	0.2	0.3	0	0.2	0.3	0.1
Absolute EOS (×10³/µL)	0	0.1	0	0.3	0.3	0	0	0.5
Absolute BASO (×10³/µL)	0	0	0	0	0	0	0	0
AST (U/L)	48	54	35	33	34	27	36	30
Alkaline Phosphatase (U/L)	339	311	317	286	296	302	296	271
Bilirubin Total (mg/dL)	0.2	0.2	0.2	0.2	0.3	0.2	0.3	0.3
Calcium (mg/dL)	9.9	9.5	9.7	9.7	9.8	9.8	9.9	9.8
Protein Total (g/dL)	7	7.1	6.7	6.9	7.2	7.3	7.2	6.9
Albumin (g/dL)	4.4	4.1	4	4.1	4.4	4.3	4.5	4.2
Glucose/Random (mg/dL)	65	88	66	95	88	81	69	87
Urea Nitrogen (mg/dL)	23	23	22	21	21	19	21	23
Creatinine (mg/dL)	0.53	0.57	0.54	0.56	0.64	0.57	0.65	0.68
Bun/ Creatinine Ratio (mgUN/mgCR)	43.4	40.4	40.7	37.5	32.8	33.3	32.3	33.8
Sodium (mmol/L)	144	143	143	144	144	143	145	145
Potassium (mmol/L)	4	3.4	3.8	3.5	2.9	3.8	3.3	3.6
Chloride (mmol/L)	104	102	103	102	101	103	104	104
Osmolality / Calc (mOsm/kg)	298	298	296	299	299	296	300	302
Carbon Dioxide (mmol/L)	18	15	27	26	21	25	22	25
Anion Gap (mmol/L)	22	26	13	16	22	15	19	16
ALT (U/L)	36	57	56	52	36	32	31	42

Supplemental methods

Plasma and tissue drug quantitation method

A Waters ACQUITY H-class UPLC (Waters, Milford, MA, USA) connected to a Xevo TQ-S micro mass spectrometer was used for UPLC-MS/MS analysis. For plasma/blood drug quantitation, 25 µL of sample was added into 1 mL acetonitrile (ACN), followed by addition of 10 µL internal standard (IS) solution to obtain a final concentration of 50 ng/ml. d3-dolutegravir (d3-DTG) and myristoylated dolutegravir (MDTG) were used as ISs for CAB and MCAB analysis, respectively. Samples were vortexed then centrifuged at 17,000 × g for 10 min. Supernatants were collected and dried using a ThermoScientific Savant Speed Vacuum (ThermoScientific, MA, USA) and reconstituted in 100 µL of 50% v/v ACN in Optima grade water; 10 µL was injected for CAB and MCAB UPLC–MS/MS analysis. Standard ACN were prepared in blank mouse plasma/blood in the range of 0.2-2000 ng/mL for CAB and MCAB. For tissue sample preparation, 15-200 mg of sample was homogenized in 4-19 volumes of 90% ACN in Optima-grade water using a TissueLyzer II (Qiagen, Valencia, CA, USA). ACN (80 µL), 50% ACN in water (10 µL), and IS (10 µL) were added to 100 µL of tissue homogenate, followed by vortexing for 3 min and centrifugation at 17,000 × g for 10 min. The supernatant (100 µL) was mixed with 50 µL of Optima grade water for sample injection. Standards were prepared similarly using blank tissue homogenates with 10 µL of spiking solution (CAB/MCAB, 5-20,000 ng/mL in 50% ACN). For CAB quantitation, chromatographic separation of 10 µL CAB sample was achieved on an ACQUITY UPLC BEH Shield RP18 column (1.7 µm, 2.1 mm x 100 mm) using a 7-min gradient of mobile phase A (7.5 mM ammonium formate in Optima grade water, adjusted to pH 3 using formic acid) and mobile phase B (100% Optima grade ACN) at a flow rate of 0.25 mL/min. The initial mobile phase composition was 35% B for the first 3.5 min and was increased to 95% B in 0.5 min and held constant for 1.5 min. Mobile phase B was then reset to 35% in 0.5 min and the column was

equilibrated for 1 min before the next injection. For MCAB quantitation chromatographic separation was achieved with a different 7-min gradient method at a flow rate of 0.35 mL/min. The initial mobile phase composition was 80% B for the first 4.5 min, and increased to 95% B in 0.25 min, held constant for 1 min, reset to 80% in 0.25 min and the column was equilibrated for 1 min before the next injection. CAB and MCAB were detected at a cone voltage of 10 V and 24 V, respectively, and a collision energy of 24 V and 18 V, respectively, in the positive ionization mode. Multiple reaction monitoring (MRM) transitions used for CAB, MCAB, d3-DTG, and MDTG were 406.04 > 126.93, 616.28 > 406.09, 422.84 > 129.99, and 630.20 > 420.07, respectively. Spectra were analyzed and quantified by MassLynx software version 4.1. All quantitations were determined using analyte peak area to internal standard peak area ratios.

Nanomedicine Production Plant Good Laboratory Practice (GLP) Facility Production of NMCAB for rhesus macaques PK study

The Operational Unit and Quality Assurance Unit work together, but independently of each other, to put into place the protocols and guidelines, which were followed during the manufacturing of NMCAB in the Good Laboratory Practice (GLP) Facility.

The Operational Unit was responsible for following approved protocols and standard operating procedures (SOPs). All of the equipment followed strict protocols for calibration and cleaning before and after use. A witness verified all procedures performed in the GLP facility and every step was initialed and dated by the operator and verifier. The operator and verifier followed specific protocols for Personal Protective Equipment (PPE) and behavior once in the GLP facility. This is a highly-restricted access facility. Due to the importance and quality of work being done only Nebraska Nanomedicine Production Plant employees are granted access. All steps of the approved protocol were documented during manufacturing.

The Quality Assurance Unit was responsible for monitoring this study to assure that the facilities, equipment, personnel, methods, practices, records, and controls were in conformance

with the regulations. The Quality Assurance Unit is entirely separate from and independent of the personnel engaged in the direction and conduct of the study. They also maintained a copy of the master schedule sheet, copies of all protocols and determined that no deviations from approved protocols or standard operating procedures were made without proper authorization and documentation.

The balances used two weight standards (10 gram and 200 gram) for calibration checks. All pipets had been calibrated. The Malvern Zetasizer Nano ZSP, which is used for size, polydispersity index and zeta potential measurements, used a 60 nm standard to check calibration. These steps were done before each use.

Equipment such as the Avestin Emulsiflex C3 High Pressure Homogenizer have cleaning protocols which were implemented before and after use. The homogenizer had at least 500 mL of methanol, then sodium hydroxide and lastly 1500 mL of Water For Injection (WFI) circulated through it before and after each manufacturing run. The removable parts of the homogenizer were placed in the oven to bake at 200 ˚C for at least 60 min. All of the glassware was also baked at 200˚ C for 60 min to remove pyrogens. The homogenizer had 40 mL of WFI run through it once it had been cleaned. A sample of this WFI was then tested for endotoxins with the Lonza Limulus Amebocyte Lysate PRYOGEN-500 test kit to verify that the cleaning procedure was successful. This process was also carried out prior to using the homogenizer to verify no growth of any endotoxin between uses.
